# Melatonin increases bone mass in normal, perimenopausal, and postmenopausal osteoporotic rats via the promotion of osteogenesis

**DOI:** 10.1186/s12967-022-03341-7

**Published:** 2022-03-16

**Authors:** Huanshuai Guan, Ning Kong, Run Tian, Ruomu Cao, Guanzhi Liu, Yiyang Li, Qilu Wei, Ming Jiao, Yutian Lei, Fangze Xing, Peng Tian, Kunzheng Wang, Pei Yang

**Affiliations:** 1 Bone and Joint Surgery Center, The Second Affiliated Hospital of Xi’an Jiaotong University, Xi’an, China; 2 Department of Gastroenterology, China-Japan Friendship Hospital, Beijing, China

**Keywords:** Melatonin, Different stages of the menopause, Osteoporosis, Osteogenesis, Bone marrow mesenchymal stem cells

## Abstract

**Background:**

Osteoporosis is a disease threatening the health of millions of individuals. Melatonin is found to be a potential anti-osteoporosis drug. However, whether melatonin plays a role against osteoporosis at different stages of the menopause and the underlying mechanisms are unknown.

**Methods:**

Ovariectomy was utilized as a model of perimenopausal and postmenopausal osteoporosis. A total of 100 mg/kg melatonin, or solvent alone, was added to the drinking water of the rats over 8 weeks. Perimenopausal rats immediately received intervention following ovariectomy while postmenopausal rats received intervention 8 weeks after ovariectomy. All rats underwent overdose anesthesia following intervention after which blood samples and femurs were collected for further analysis. Rat femurs were scanned using micro-CT and examined histologically. The serum levels of melatonin and osteogenic biochemical markers were measured and the expression of osteogenesis-associated genes (*Runx2*, *Sp7*) were quantified by real-time quantitative PCR. Alkaline phosphatase (ALP) activity and the gene expression (*Col1a1*, *Runx2*, *Alpl*, and *Bglap*) were measured after bone marrow mesenchymal stem cells (BMSCs) were osteogenically induced, both with and without melatonin in vitro. ALP staining and Alizarin Red S staining were used to identify osteogenesis.

**Results:**

Analysis by micro-CT and histological staining demonstrated that bone mass decreased and bone microarchitecture deteriorated over time after ovariectomy. Intervention with melatonin increased bone mass in normal, perimenopausal, and postmenopausal osteoporotic rats. Serum levels of ALP continuously increased after ovariectomy while osteocalcin levels initially rose, then decreased. Melatonin increased the serum levels of ALP and osteocalcin and mRNA expression levels of *Runx2* and *Sp7* in normal and postmenopausal rats, the opposite of the markers in perimenopausal rats. In vitro study demonstrated that 100 μmol/L melatonin increased the mRNA expression of *Col1a1*, *Runx2*, and *Alpl* three and/or seven days after intervention, and *Alpl* and *Bglap* 14 d after intervention. Melatonin increased ALP activity and the extent of ALP and matrix mineralization in the late stage of osteogenesis.

**Conclusions:**

Bone mass continuously decreased after ovariectomy, while melatonin increased bone mass and ameliorated bone metabolism in normal, perimenopausal, and postmenopausal osteoporotic rats due to the induction of osteogenic differentiation in BMSCs.

## Background

Osteoporosis (OP) is a progressive metabolic bone disease in which systemic bone mineral density (BMD) decreases and bone microarchitecture deteriorates due to the imbalance between bone formation and resorption. Because of the increased fragility of such bone, there is a dramatic increase in the risk of bone fractures, occurring mostly in the wrist, hip, and lumbar spine, representing a serious health threat to a large number of individuals, particularly postmenopausal women and the elderly [1]. OP is considered among the most serious public health concerns, representing a considerable financial burden as the worldwide population ages. Almost 48 million Americans over the age of 50 suffer from osteopenia and 9 million have OP, according to data from 2013, with projections for 64.3 million osteopenic patients and 11.9 million OP patients by 2030 if no specific intervention is administered. Of the different pathological types of OP, postmenopausal OP is the most common, affecting more than 30% of elderly women [2]. However, current clinical anti-osteoporosis drugs such as bisphosphonate and denosumab have multiple disadvantages, including serious side effects, poor efficacy, a high price, and a long time course that results in poor patient compliance [2, 3]. Consequently, novel anti-osteoporosis drugs with greater efficacy and fewer disadvantages must be developed.

Melatonin (MT) is an indoleamine, also known as N-acetyl-5-methoxytryptamine, predominantly secreted by the pineal gland and present in almost all parts of the bodies of mammals [4]. Moreover, MT can be produced in small quantities in the spleen, thymus, testis, retina, gastrointestinal tract, and bone marrow [5]. The production of MT is synchronized with the light/dark cycle and specifically, is synthesized and secreted in dark environments or where light is very limited [6]. Studies have demonstrated that MT is involved in the synchronization of the circadian clock in peripheral tissues and so can be used to treat insomnia [4]. As an amphiphilic chemical messenger, MT is widely involved in seasonal timing, sleep, wake cycle control, and energy metabolism [7]. Furthermore, MT exerts numerous physiological effects, such as inhibition of inflammation, oxidation, tumors, and providing cardiovascular and neurological protection and immune regulation [5, 7–11].

MT participates in the maintenance of bone and cartilage and is closely associated with bone metabolic homeostasis. It has been found that MT is synthesized and secreted by bone marrow tissue [5] and is in high concentration in the femoral bone marrow [12]. As there is a relationship between the occurrence of OP with aging and the menopause, which is accompanied by a reduction in the secretion of MT, researchers have found that there is a correlation between reduced plasma MT levels and an increased incidence of bone deterioration [13, 14]. Furthermore, bone metabolism biomarkers, such as serum alkaline phosphatase (ALP) increase after removal of the pineal gland while exposure to MT reduces them [15, 16]. In vivo studies have demonstrated that bone loss can be restored by MT in postmenopausal females and estrogen-deficient mice and rats [17–20]. Moreover, MT can attenuate the bone loss induced by retinoic acid [3] and type 2 diabetes [21] and save the bone mass in naturally aged mice [22]. These studies suggest the possibility of MT in the treatment of OP. In addition, few severe side effects involving MT have been identified so far, while minor problems, such as insomnia, can be solved in other ways [12]. Above all, considering that it is inexpensive, has a broad positive impact on tissues with almost no side effects, MT should be considered a potential treatment strategy for OP.

Although some studies of the effectiveness of MT on menopausal OP have been published, researchers generally have administered MT at the time of ovariectomy (OVX) [17, 19, 23, 24], or after OP had already become established [20, 25]. Systematic analysis of changes in bone mass and bone metabolism with time after OVX was lacking and the effects of MT in different stages of the menopause are still unknown. The concrete mechanisms by which MT reverses bone loss remain unclear. We have investigated both stages in the present study and explored potential mechanisms. We first explored the impact of the menopause in rats and then investigated the hypothesis that MT could reverse bone loss and ameliorate bone metabolism at both early and late stages. Furthermore, we investigated whether MT increased osteogenesis of bone marrow mesenchymal stem cells (BMSCs) at different time points of osteogenic differentiation.

## Methods

### Animals and OVX

The institutional Animal Care and Use Committee of Xi’an Jiaotong University approved all animal surgical and experimental procedures. A total of 48 twelve-week-old female Sprague–Dawley rats weighing 300 ± 20 g were purchased from the Animal Center of the Medical School of Xi’an Jiaotong University and housed with four rats per cage within specific-pathogen-free conditions (22–24 °C, 50–60% humidity, and 12 h:12 h light/dark cycle). The animals were provided ad libitum access to water and a standard laboratory rodent diet. After a 1-week period of acclimatization, the rats were allocated randomly into 6 groups (Table 1): sham + solvent control group (A: Sham + Ctrl), sham + MT group (a: Sham + MT), early OVX + solvent control group (B: Early + Ctrl), early OVX + MT group (b: Early + MT), late OVX + solvent control group (C: Late + Ctrl), late OVX + MT group (c: Late + MT). At specified time points, a rat model of OP was induced in the model groups using bilateral OVX, while the other groups received sham surgery. Briefly, the rats were anesthetized by intraperitoneal injection of 30 mg/kg sodium pentobarbital (Sigma-Aldrich, Missouri, USA) and placed in a prone position. The skin over the middle section of the spine was shaved and then disinfected. After local lidocaine infiltration anesthesia, a 3 cm longitudinal incision was created after which the fascia and muscles were bluntly separated. After exploration, fat tissue in which each ovary was located, was then extracted. In the middle of each fallopian tube, the ovarian blood vessels were ligated and cut, while the ovaries of sham groups were left intact. All remaining tissue was placed back and the surgical incision was then sutured together layer-by-layer. The rats were then placed in cages to recuperate. The rats were administered a total of 80,000 U sodium penicillin (North China Pharmaceutical Co Ltd, Hebei, China) intramuscularly daily for 3 consecutive postoperative days. MT (Aladdin, Shanghai, China) was dissolved in ethanol to prepare a stock solution. In the appropriate groups, drinking water was administered in brown bottles containing approximately 100 mg/kg MT final concentration 0 w (early group) or 8 w (late group) after surgery for a total of 8 w. After intervention, the rats received overdose anesthesia. Blood and the bilateral femurs were harvested, processed, and then stored at − 80 °C or in 4% paraformaldehyde (PFA; Servicebio, Wuhan, China) for additional testing.

**Table 1:** Animal study groups

Group		Quantity	OVX	MT
Sham	A: Sham + Ctrl	8	−	−
	a: Sham + MT	8	−	+
Early	B: Early + Ctrl	8	+	−
	b: Early + MT	8	+	+
Late	C: Late + Ctrl	8	+	−
	c: Late + MT	8	+	+

### Enzyme-linked immunosorbent assay (ELISA)

Each blood sample was centrifuged at 3000*g* for 15 min and the serum supernatant was removed and placed in a fresh centrifuge tube. The levels of MT, ALP, and osteocalcin (OCN) were quantified using the respective ELISA kits (Jianglai, Shanghai, China), in accordance with the manufacturer’s instructions. Absorbance at 450 nm was measured using a microplate reader (S/N 415–2687, Omega Bio-Tek, Ortenberg, Germany).

### Serum biochemical tests

To determine the influence of MT on biochemical indices, calcium and phosphorus serum levels were quantified using an automatic biochemical analyzer (Chemray 800, Rayto Life and Analytical Sciences Co. Ltd, Shenzhen, China).

### Micro-CT assessment

After fixation in 4% PFA for 3 days, the microarchitecture of the distal femur was examined non-destructively using a Y. Cheetah micro-CT system (Yxlon, Hamburg, Germany). Briefly, the left femurs were fixed and scanned at a voltage of 80 kV and current of 62.5 mA at high resolution (10 μm). Three-dimensional (3D) reconstruction and analysis were performed using VG Studio MAX 3.0 software (Volume Graphics, Heidelberg, Germany). Cancellous and cortical bone 1 mm from the growth plate of the distal metaphysis was defined as a region of interest. To evaluate bone mass and microstructure, the bone volume ratio (BV/TV), bone surface/volume ratio (BS/BV), trabecular separation/spacing (Tb.Sp), trabecular number (Tb.N), trabecular thickness (Tb.Th), and cortical thickness (Ct.Th) were calculated and analyzed.

### Histopathological analysis

After micro-CT evaluation, distal portions of the left femurs were removed then decalcified in 13% ethylenediaminetetraacetic acid (EDTA, Servicebio, Wuhan, China). The decalcification solution was refreshed every two days for 4 weeks. The bones were then dehydrated through an increasing gradient of ethanol concentrations, cleared in xylene, then finally embedded in paraffin wax. The blocks were sliced sagittally into 5 μm sections using a microtome. Hematoxylin–eosin (HE), Goldner trichrome, and Safranin O-Fast Green staining were performed on the sections for analysis of the bone trabecular and cartilage structure, in accordance with standard procedures. The sections were scanned using a Nanozoomer pathology slide scanner (Hamamatsu Photonics, Shizuoka, Japan) then viewed using NDP.view2 software. ImageJ software (National Institutes of Health, Bethesda, USA) was used to quantify all histological sections. All sections were analyzed by 2 observers blinded to the study.

### Cell culture

C57Bl/6 J mouse BMSCs were purchased from Procell Life Science & Technology Co. Ltd (Wuhan, China) and cultured in Dulbecco’s modified Eagle’s medium (DMEM; Gibco, New York, USA) supplemented with 10% fetal bovine serum (FBS; Gibco, New York, USA), 1% penicillin–streptomycin (Gibco, New York, USA) at 37 °C/5% CO_2_. When the cell cultures were 80–90% confluent, they were trypsinized using 0.25% trypsin–EDTA (Gibco, New York, USA). The cells were then washed with phosphate-buffered saline (PBS) then plated in 25 cm^2^ culture flasks at passage 1, at a ratio of 1:4. The cells were maintained at 37 °C within a humidified atmosphere containing 5% CO_2_. The culture medium was refreshed every three days. Cells from passages 3 to 6 were used in subsequent experiments.

### Cell proliferation assay

To determine whether MT influences the proliferation of BMSCs and identify the optimal intervention concentration, a cell counting kit-8 assay (CCK-8; Dojindo Laboratories, Kumamoto, Japan) was used, in accordance with the manufacturer’s instructions. BMSCs in 100 μL DMEM supplemented with 10% FBS, 100 U/mL penicillin, and 100 μg/mL streptomycin were seeded in 96-well plates (1 × 10^3^ cells/well) and administered MT (Sigma-Aldrich, Missouri, USA) at concentrations of 0, 10^–7^, 10^–6^, 10^–5^, 10^–4^, 10^–3^, and 10^–2^ mol/L. At specified time points, the medium was removed and 100 μL DMEM containing 10 μL CCK-8 solution was added to each well, after which the plates were incubated at 37 ℃/5% CO_2_ for 1–2 h. Absorbance at 450 nm was measured using a microplate reader (S/N 415–2687, Omega Bio-Tek, Ortenberg, Germany) from which optical density (OD) values were recorded. The assays were performed at 0 h, 24 h, 48 h, 72 h, and 96 h after intervention. The experiments were repeated three times.

### Osteogenic differentiation

BMSCs were seeded in triplicate in 6 or 12-well plates and cultivated until 60–70% confluent. Osteogenic induction medium comprising DMEM, 10% FBS, 0.1 μmol/L dexamethasone, 10 mmol/L β-glycerophosphate, and 50 μg/mL ascorbic acid with or without 100 μmol/L MT was added to induce osteogenic differentiation. The medium was replaced every 3 days with fresh osteogenic medium until the ALP activity was measured. Cells were stained for ALP and with Alizarin Red S (ARS), and RNA was isolated from other populations of the cells.

### ALP activity measurement

ALP assay kits (Beyotime Biotech, Shanghai, China) were used to measure ALP activity in the BMSCs at 3, 7, and 14 d after osteogenic differentiation. Briefly, the culture medium was removed and the cells were washed in PBS, after which 100 μL Radio Immunoprecipitation Assay buffer (RIPA; Beyotime Biotech, Shanghai, China) was added to each well for 10 min and the mixture centrifuged at 12,000*g* for 10 min. The supernatant was removed to fresh centrifuge tubes and ALP activity was measured in accordance with the manufacturer’s instructions.

### ALP staining

BCIP/NBT alkaline phosphatase color development kits (Beyotime Biotech, Shanghai, China) were used to measure the expression of ALP in BMSCs, in accordance with the manufacturer’s instructions, after 3, 7, and 14 d of osteogenic differentiation. Briefly, slides on which cells were grown were washed with PBS, fixed in 4% PFA for 30 min, then washed again with PBS prior to incubation in the dark with BCIP/NBT staining solution for 1–2 h at room temperature, then washed with double distilled water (ddH_2_O). Finally, the slides were observed using light microscopy (ECLIPSE Ts2, Nikon, Tokyo, Japan). At least three replicates were performed in each group for comparison.

### ARS staining

Cell mineralization was measured using ARS staining solution (Beyotime Biotech, Shanghai, China) 14 or 21 days after osteogenic differentiation. Briefly, the cells were rinsed with PBS, fixed in 4% PFA for 30 min, then stained using ARS solution for 30 min at 37 °C after washing three times with ddH_2_O, as per the manufacturer’s instructions. Images of stained cells were then acquired using a light microscope, following three washes with PBS. The experiment was performed in triplicate. Quantification of ALP and Alizarin Red S staining were conducted using ImageJ software.

### Real-time quantitative PCR (RT-qPCR)

Total RNA from BMSCs and blood samples was extracted using Trizol reagent (Takara, Dalian, China) or Trizol LS reagent (Invitrogen, California, USA), in accordance with the manufacturer’s instructions. Total RNA concentration was measured using a Nanodrop 2000 spectrophotometer (Thermo Fisher Scientific, Massachusetts, USA). cDNA was then synthesized from 1 μg total RNA using a StarScript II First-strand cDNA synthesis mix (GenStar, Beijing, China), in accordance with the manufacturer’s protocols. Briefly, 1 μL StarScript II RT Mix, 10 μL 2 × RT reaction mix, and 1 μg RNA and diethylpyrocarbonate-ddH_2_O (DEPC-ddH_2_O) in a volume of 20 μL were mixed and reacted at 42 °C for 15 min, after which the reaction was finally terminated by raising the temperature to 85 °C for 5 min. As previously described[26], RT-qPCR was conducted using 2 × Universal SYBR Green Fast qPCR mix (ABclonal, Wuhan, China) in a final volume of 20 μL, comprising 10 μL mix, 0.8 μL primer, 1 μL cDNA, and 8.2 μL DEPC-ddH_2_O within a thermocycler. The thermocycling conditions were as follows: 3 min at 95 °C, then 42 cycles of 5 s at 95 °C and 32 s at 60 °C. All assays were conducted in triplicate in a Bio-Rad system (CFX Connect, California, USA). Cycle threshold (Ct) values were recorded for each gene and the expression levels of target mRNAs were calculated using the standard 2^−△△Ct^ method. Glyceraldehyde 3-phosphate dehydrogenase (Gapdh) was used as the endogenous control. The primer sequences of the genes analyzed in the present study are listed in Table 2.

**Table 2:** Primers for RT-qPCR

Gene	Forward primer	Reverse primer
Rat-Gapdh	GCAAGTTCAACGGCACAG	CGCCAGTAGACTCCACGAC
Rat-Runx2	CTTCGTCAGCGTCCTATC	CTTCCATCAGCGTCAACA
Rat-Sp7	CTTCTCAAGCACCAATGGA	CTAGGCAGGCAGTCAGAA
Mouse-Gapdh	TGTTTCCTCGTCCCGTAG	CAATCTCCACTTTGCCACT
Mouse-Col1a1	ACCTCCCAGTGGCGGTTATGAC	AGTTCTTCTGAGGCACAGACGG
Mouse-Alpl	ATCTTTGGTCTGGCTCCCATG	TTTCCCGTTCACCGTCCAC
Mouse-Runx2	TTTGCAGTGGGACCGACA	AGCCATGGTGCCCGTTAG
Mouse-Bglap	GCAGGAGGGCAATAAGGT	GCTGATATGCGATGTCCTT

*Gapdh* glyceraldehyde 3-phosphate dehydrogenase, *Runx2* RUNX family transcription factor 2, *Sp7* Sp7 transcription factor, *Col1a1* Collagen type I alpha 1 chain, *Alpl* alkaline phosphatase, *Bglap* bone gamma carboxyglutamate protein

### Statistical analysis

All results are presented as means ± standard error of the mean (SEM) of three independent experiments. The data were statistically analyzed using an unpaired Student’s t-test of independent samples. *P* < 0.05 was considered statistically significant. IBM SPSS Statistics 24 (IBM, New York, USA) and GraphPad Prism 8 (GraphPad, San Diego, USA) software were used to conduct all statistical analyses.

## Results

### The rat postmenopausal OP model was successfully established, with bone mass decreasing over time after OVX

**Fig. 1:**
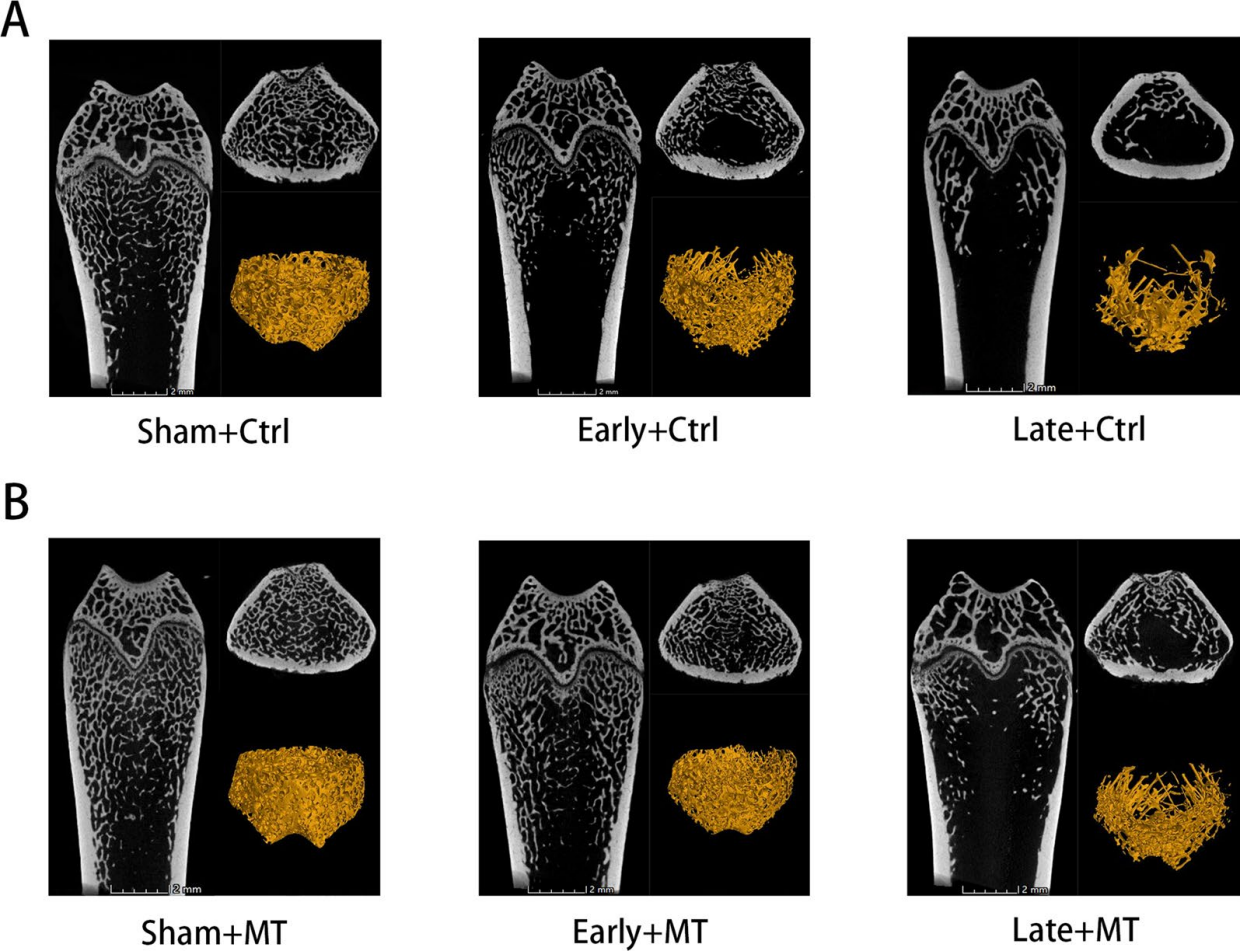
Micro-CT images of femurs (coronal position, transverse position, and 3D reconstruction of trabecular bone) in the control (**A**) and MT (**B**) groups. **A** Decreased trabecular bone and porosity of the trabecular bone structure were observed in the OVX groups, with bone mass decreasing over time. **B** Compared with the control groups, MT increased the quantity of trabecular bone and ameliorated the porosity of the trabecular bone structure in the sham and OVX groups. n = 3 per group

**Fig. 2:**
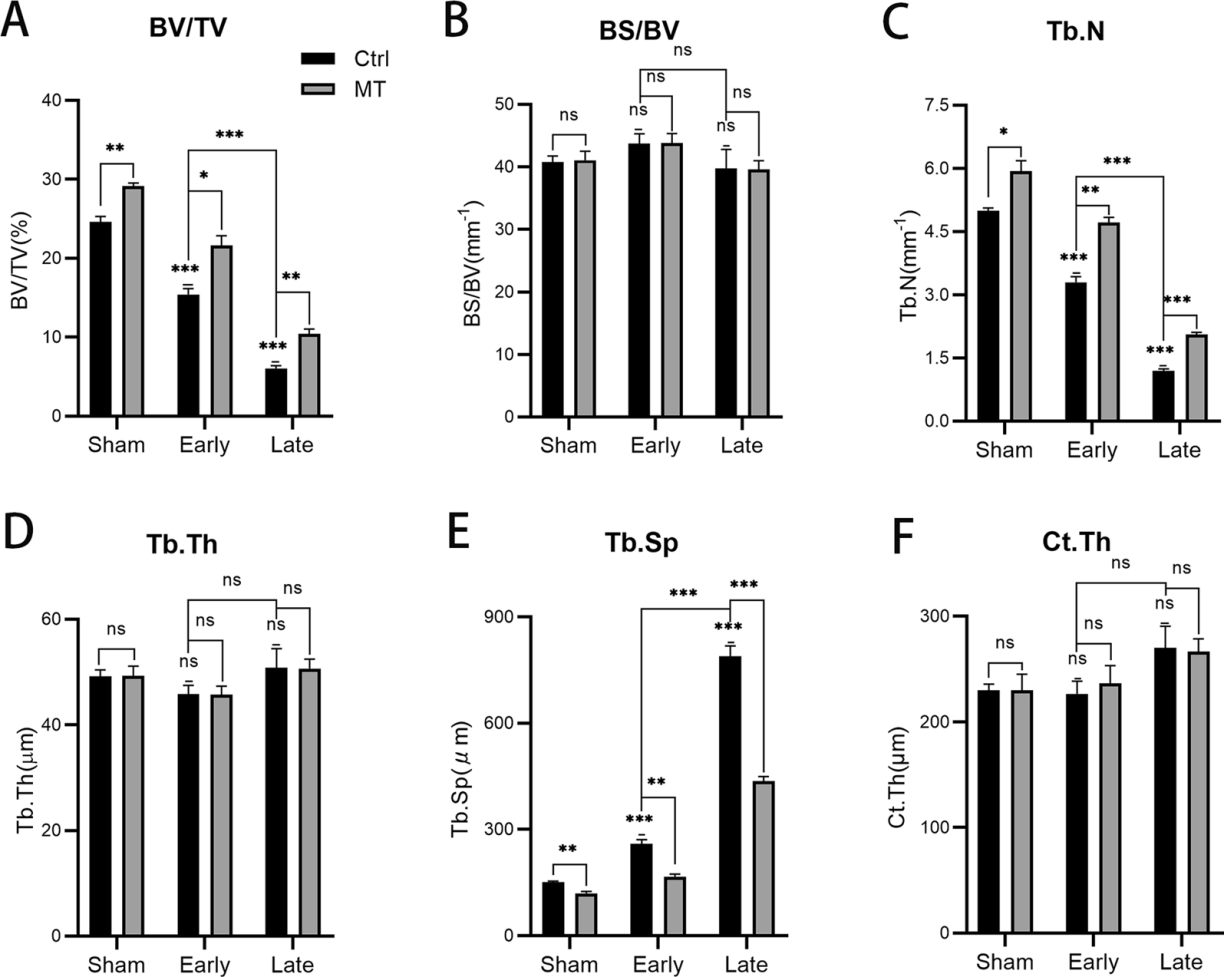
Micro-CT parameters, including BV/TV (**A**), BS/BV (**B**), Tb.N (**C**), Tb.Th (**D**), Tb.Sp (**E**), and Ct.Th (**F**) in all groups. n = 3 per group. The data represent means ± SEM. For the single statistical markers above, the early and late groups were compared with the Sham + Ctrl group. Unpaired t-tests were used to compare groups. ns represents no statistical difference, *P < 0.05, **P < 0.01,  ***P < 0.001

As OP is principally characterized by a decrease in BMD and deterioration of the bone microarchitecture, we firstly confirmed that the rat OP model was established correctly from the bone microstructure using micro-CT. The results (Figs. 1A, 2) demonstrated that, in addition to the quantity of trabecular bone loss in the OP model group, the mean width of the medullary cavity between the trabecular bones increased and the BMD was low, validating that the postmenopausal OP rat model was successfully constructed. Specifically, both BV/TV (37.31%) and Tb.N (34.00%) exhibited a significant decrease in values in the early OVX group, while Tb.Sp (72.12%) increased significantly compared with the sham group. Unless specifically mentioned, the sham, early OVX, and late OVX groups describe the sham + control, early OVX + control, and late OVX + control groups. In addition, more significant bone mass loss and decreased trabecular bone number were supported by lower BV/TV (75.39%) and Tb.N (76.20%) values and a higher Tb.Sp value (422.32%) in the late OVX group compared with the sham group, indicating that postmenopausal OP would progress if untreated. No statistical difference was observed between the sham, early OVX, and late OVX groups in terms of BS/BV, Tb.Th, or Ct.Th. The results indicate that the postmenopausal OP rat model had been successfully established, with bone mass decreasing over time following OVX.

### MT increased the quantity of trabecular bone and ameliorated the porosity of the trabecular bone structure in all groups

The effect of MT on bone mass and bone microstructure was analyzed using micro-CT. As shown in the micro-CT images (Figs. 1B, 2), MT slightly improved bone mass and the quantity of trabecular bone in the sham group. Specifically, the BV/TV (18.71%) and Tb.N (18.80%) values increased slightly, while Tb.Sp (20.73%) decreased slightly compared with the control group. However, it was more significant that intervention with MT was more effective in improving the quantity of trabecular bone and ameliorated the porosity of the trabecular bone structure in the OVX groups. The results indicate that the rats in the OVX groups treated with MT had higher BV/TV and Tb.N values and lower Tb.Sp values compared with the controls in the early (BV/TV: 40.17%, Tb.N: 42.73%, Tb.Sp: 35.82%) and late groups (BV/TV: 72.40%, Tb.N: 73.11%, Tb.Sp: 44.68%). Furthermore, the BS/BV, Tb.Th, and Ct.Th values did not change significantly following MT intervention. The results demonstrate that MT intervention increased the quantity of trabecular bone and ameliorated the porosity of the trabecular bone structure in all groups.

### Histological analysis of rat bone tissue

**Fig. 3:**
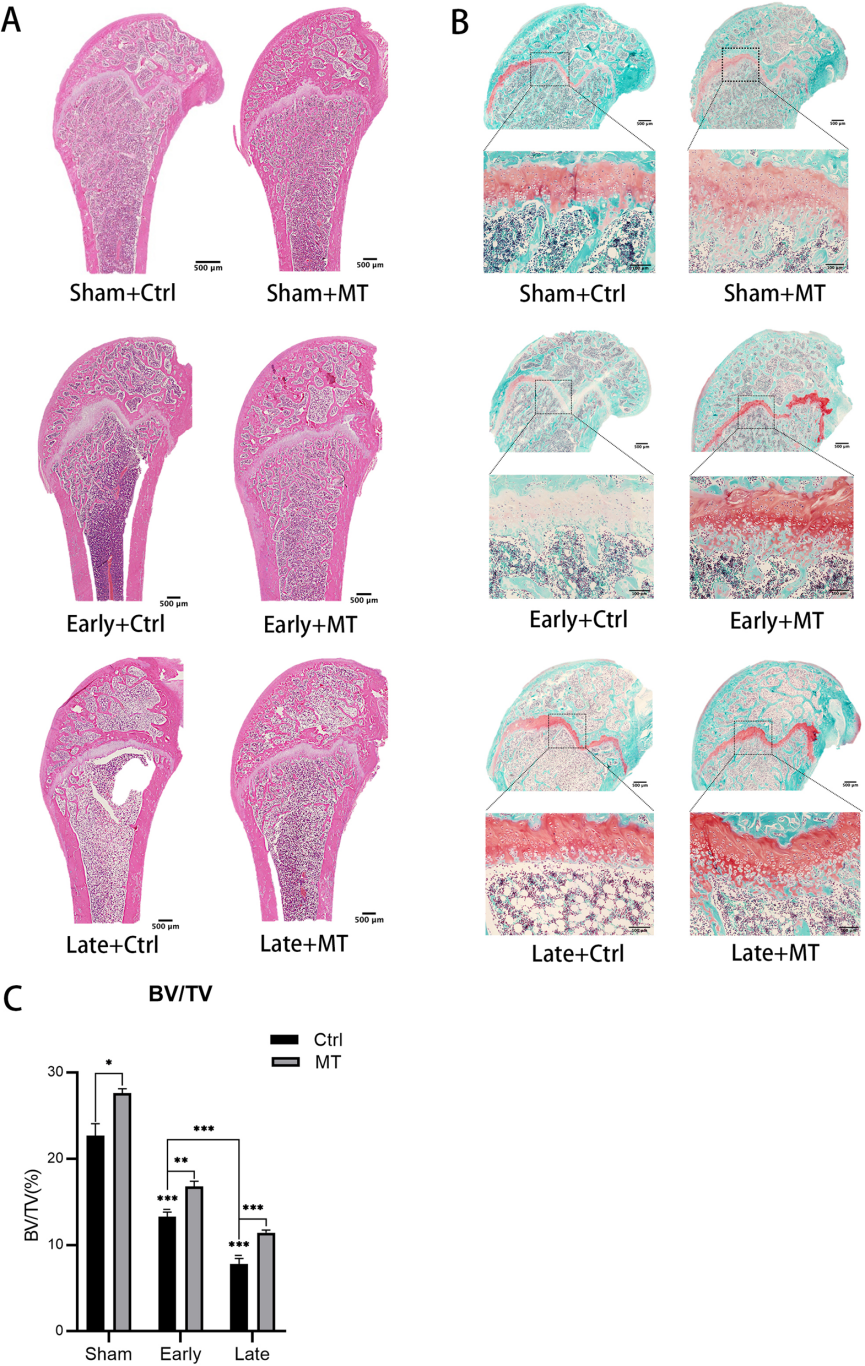
HE staining (Sagittal position) (**A**), Safranin O and Fast Green staining (Sagittal position) (**B**), and histomorphometric analysis (**C**) of femurs and the effect of MT. n = 4 per group. Data represent means ± SEM. For the single statistical markers above, the early and late groups were compared with the Sham + Ctrl group. Unpaired t-tests were used to compare groups. *P < 0.05, **P < 0.01, ***P < 0.001

Consistent with the results of micro-CT, HE staining further confirmed the success of the OP model and the bone protective effects of MT on OVX rats (Fig. 3A). Histomorphometric analysis (Fig. 3C) confirmed a decrease in bone volume in the early OVX (41.47%) and late OVX groups (65.76%) compared with the sham group. MT increased the bone volume in the sham (21.64%), early OVX (26.28%), and late OVX groups (46.98%) compared with each control group. It is clear that MT played a more significant role in the late OVX group, where rats were experiencing OP. Inspection of the metaphysis of the distal femur of the rats by Safranin O and Fast Green staining (Fig. 3B), demonstrated that epiphyseal cartilage, endochondral ossification, and some primary trabeculae were clearly present in the sham group, while endochondral ossification and primary trabeculae gradually decreased in the early and late OVX groups and almost undetectable in the late OVX group. MT treatment increased the extent of endochondral ossification and the number of primary trabeculae compared with the early OVX and late OVX groups.

### Effects of MT on serum biochemical metabolism

#### Oral administration of MT increased its concentration in blood

**Fig. 4:**
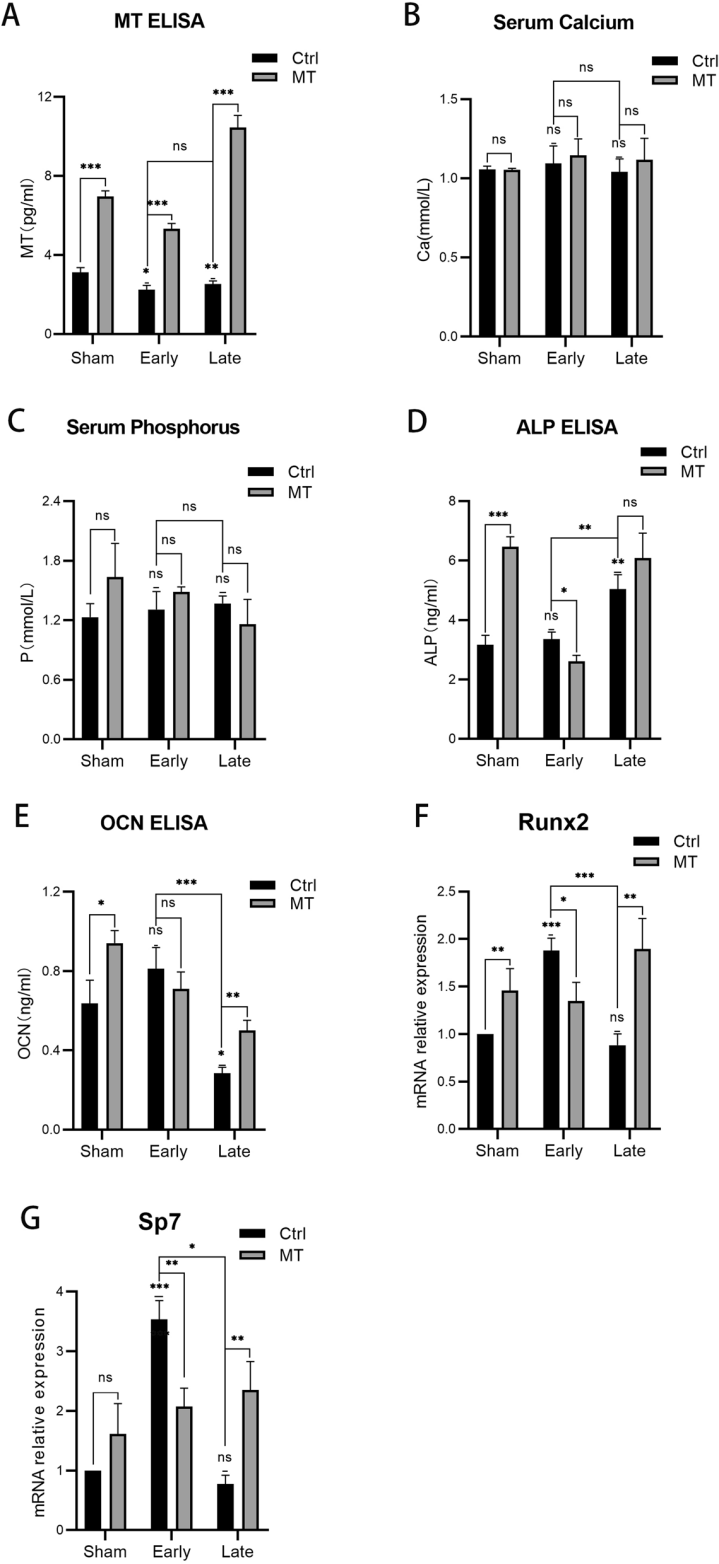
Levels of serum MT (**A**), calcium (**B**), phosphorus (**C**), ALP (**D**), OCN (**E**), and gene expression levels of *Runx2* (**F**) and *Sp7* (G). n = 3 per group. Data represent means ± SEM. The single statistical markers above in the early and late groups were compared with the Sham + Ctrl groups. Unpaired t-tests were used to compare groups. ns represents no statistical difference, *P < 0.05, **P < 0.01, ***P < 0.001

Serum MT levels were quantified using ELISA to determine whether oral administration of melatonin via drinking water would maintain high levels of MT in the blood. MT levels in the early and late OVX groups were lower than in the sham group (Fig. 4A), while in all groups in which MT was administered, a clearly higher concentration of MT was observed compared with each control group (Sham: 123.08%; Early: 137.17%; Late: 313.11%), confirming that oral administration was effective.

#### Changes in serum calcium and phosphorus

The levels of serum calcium and phosphorus were quantified using an automated biochemical analyzer. The results (Fig. 4B, C) demonstrate that no significance among the sham, early OVX, and late OVX groups was observed for calcium and phosphorus levels, while after MT intervention, no statistical differences were observed between the three groups compared with each control group.

#### Changes in serum biochemical markers of bone metabolism

The levels of serum ALP and OCN were measured by ELISA, as presented in Fig. 4D, demonstrating that in the control groups, ALP levels in the sham, early OVX, and late OVX groups gradually increased, although statistical differences failed to be observed only between the sham and early OVX groups. Treatment with MT resulted in an increasing trend in ALP levels in the sham (*P* < 0.001) and late OVX groups, while ALP levels decreased after intervention in the early OVX group. OCN levels (Fig. 4E) in the early OVX group were higher than in the sham group, although not significantly so, while the levels decreased in the late OVX group. The observations were mirrored with the ALP levels, levels increasing after MT intervention in the sham (*P* < 0.05) and late OVX (*P* < 0.01) groups while decreasing in the early OVX group (but not significantly so).

#### Expression of osteogenesis-associated genes

The expression in blood of osteogenic-related markers such as *Runx2* and *Sp7* were measured (Figs. 4F, G), both increasing in the early OVX group but decreasing in the late OVX group (not significantly so). MT intervention promoted the expression of *Runx2* and *Sp7* in the sham and late OVX groups, although the expression levels decreased in the early OVX group.

### MT promoted the osteogenic differentiation of BMSCs

#### Effects of MT on cell proliferation

**Fig. 5:**
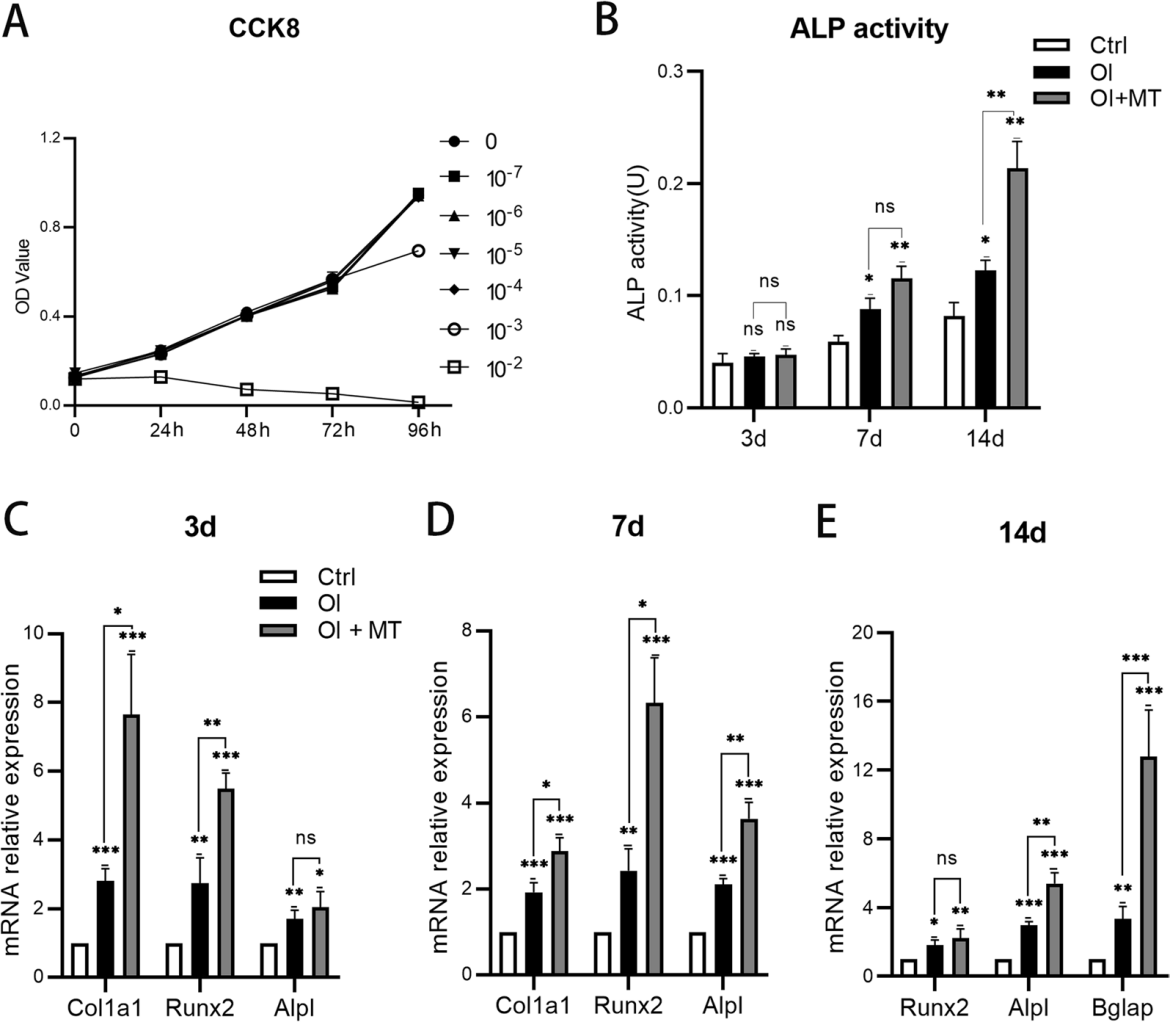
Statistical analysis of CCK-8 assay (**A**), ALP activity (**B**), expression of osteogenic genes after 3 d (**C**), 7 d (**D**) and 14 d (**E**) after intervention with MT. n = 3 per group. Data represent means ± SEM. The single statistical markers above in the OI and OI + MT groups were compared with the Ctrl groups. Unpaired t-tests were used to compare groups. ns represents no statistical difference, *P < 0.05, **P < 0.01, ***P < 0.001

The influence of MT on BMSCs was determined using a CCK-8 cell proliferation assay with six concentrations of MT from 10^–7^ to 10^–2^ mol/L. The results (Fig. 5A) demonstrate that MT did not influence the proliferation of BMSCs at concentrations of 10^–7^–10^–4^ mol/L compared with the control. However, 10^–3^ mol/L MT inhibited cell proliferation after 96 h while it was clearly inhibited by 10^–2^ mol/L MT from 24 h. Therefore, 10^–4^ mol/L MT (100 μmol/L) was selected as the intervention concentration to avoid any influence on normal cell proliferation in subsequent experiments.

#### MT increased ALP activity

ALP activity was measured using a commercial kit. No statistical differences were observed between the control group (Ctrl), osteogenesis induction group (OI), and osteogenesis induction + MT group (OI + MT), 3 d after induction (Fig. 5B). ALP activity was promoted in the OI and OI + MT groups 7 d after induction. Compared with the OI group, MT intervention significantly enhanced ALP activity 14 d after induction. The results indicate that MT promoted ALP activity during the osteogenic induction of BMSCs.

#### MT increased the expression of osteogenesis-associated genes

The expression of osteogenesis-associated genes was quantified, the results (Fig. 5C–E) demonstrating that the gene expression levels clearly increased in the OI group compared with the control, indicating that the osteogenic induction medium was effective. Moreover, 100 μmol/L MT enhanced the expression of *Col1a1* and *Runx2* three and seven days after intervention. The expression of *Alpl* increased after MT intervention but not significantly so. The expression of *Bglap* was significantly greater 14 d after MT intervention. The results indicate that MT promoted the expression of osteogenic-related genes and facilitated the osteogenic differentiation of BMSCs.

#### MT promoted the osteogenic differentiation of BMSCs and matrix mineralization

**Fig. 6:**
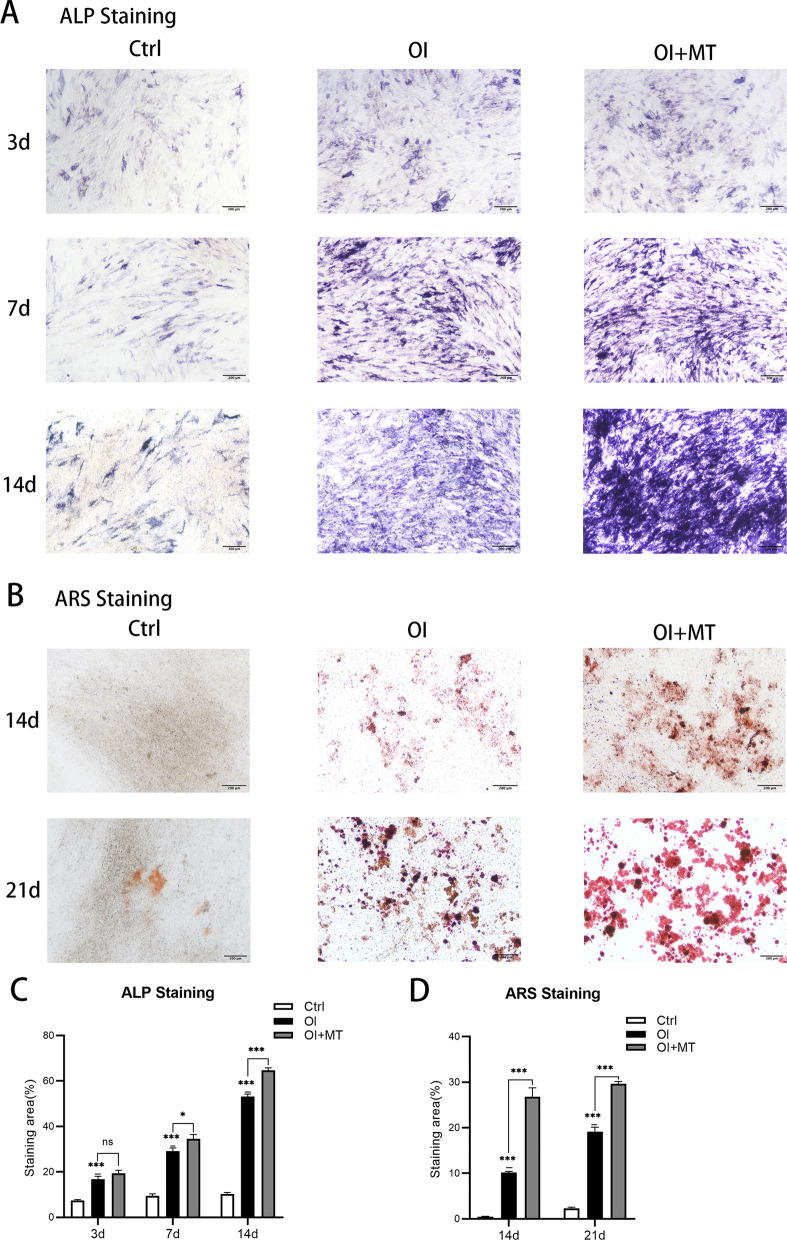
ALP (**A**), ARS (**B**) staining and statistical analysis of ALP (**C**) and ARS (**D**) staining after osteogenic induction with MT. n = 3 per group. Data represent means ± SEM. The single statistical markers above in the OI and OI + MT groups were compared with the Ctrl groups. Unpaired t-tests were used to compare groups. ns represents no statistical difference, *P < 0.05, **P < 0.01, ***P < 0.001

The results (Fig. 6A) indicated that following induction of osteogenic differentiation, the quantity of ALP and extent of matrix mineralization were both clearly promoted compared with the control. After 3 d, 7 d, and 14 d of osteogenic induction, ALP staining in the MT group was significantly darker than in the OI group. As displayed in figure (Fig. 6B), a greater number of calcium nodules were observed in the MT group by ARS staining 14 d and 21 d after osteogenic induction compared with the OI group, suggesting that matrix mineralization had been facilitated. Quantitative analysis (Fig. 6C, D) indicates that there were no significant differences between the OI and MT groups in the 3 d ALP staining. However, after 7 d and 14 d (ALP staining), and 14 d and 21 d (ARS staining), significant differences between the OI and OI + MT groups were observed.

## Discussion

OP is a progressive metabolic bone disease resulting in hip and vertebral fractures, leading to a substantial burden on both individuals and society [27]. However, the precise etiology and pathogenesis of OP remain unclear and drugs currently prescribed clinically, such as bisphosphonate, raloxifene, and teriparatide, cause side effects that result in adverse effects that cannot be ignored [2, 3]. Therefore, the development of novel drugs that could prevent or delay the progression of OP remains the focus of research programs in OP.

The rescue of OP by MT has previously attracted the attention of researchers. It has been found that MT can enhance the microstructure and biomechanical properties of bone in 22-month-old male Wistar rats [28], reduce bone loss in a dose-dependent manner in female OVX C57BL/6 J mice [19], and prevent structural and functional degeneration of bone in C57BL/6 J female mice 6 w after OVX [20]. OVX is considered the “gold standard” model for the study of postmenopausal OP, with hypoestrogenism induced by OVX replicating the principal characteristics of menopause [29]. Evident bone loss and deterioration of the bone microstructure have been observed 8 weeks after OVX [19]. However, in existing studies, researchers have selected either animals in the perimenopausal phase (immediately after OVX) or those which had clearly established postmenopausal osteoporosis (ovariectomized a period of time earlier, such as 8 weeks). A comprehensive study of the effects of MT at different stages of the menopause is clearly required. Thus, we have defined groups in which MT was administered to rats just after OVX as the perimenopausal group (early group) and in groups in which MT was administered 8 weeks after OVX as the postmenopausal osteoporotic group (late group). In the present study, we first established the quality of bone and its metabolism in rats in the normal, perimenopausal, and clearly osteoporotic phases, clarifying the effects of menopause on rats. The effects of MT on bone-related parameters in rats in the three phases were explored, in a natural and minimally invasive form of administration, namely orally.

In the present study, classic OVX was selected as a model of postmenopausal OP. Micro-CT and histological staining were used to analyze the microarchitecture of bone. The rats exhibited apparent bone mass loss and deterioration in bone microarchitecture 8 weeks after OVX, indicating that the model was successfully established. Moreover, the condition of the bone was severe 16 weeks after OVX, indicating that bone loss would progress in postmenopausal OP if it remained untreated. After OVX, endochondral ossification and the number of primary trabeculae gradually decreased, indicating that new bone formation decreased over time, which is similar to naturally aged mice [22]. The observations corroborated an earlier report [30] that levels of serum MT decreased after menopause which may be associated with changes of bone metabolism, while serum calcium and phosphorus displayed no differences. It was interesting that serum concentrations of ALP and OCN increased slightly (not statistically so) in the early OVX stage, with the level of ALP continuing to increase and the level of OCN exhibiting the opposite trend in the late stage of OVX. Levels of hormones including estrogen exhibited a dramatic change during the perimenopausal phase [31], interfering with bone metabolism and the pathway promoting osteogenesis becoming initiated after the start of bone loss. Fluctuations in hormones and the initiation of the bone-promoting program work together, resulting in changes in serum ALP and OCN in the early OVX stage. High levels of ALP, an early marker of bone formation, were generated as a result of persistent bone loss in the late OVX stage. Moreover, ALP can also be affected by hepatic metabolism, which may affect the results as a result of the decrease of estrogen. However, decreased mineralization capacity caused OCN, a late marker of bone formation unable to manage this change, thus, the decreased levels of OCN indicated poor osteogenic capability in the late OVX stage. The mRNA expression of osteogenic genes (*Runx2*, *Sp7*) increased significantly during perimenopausal periods as a result of initial bone loss and then, decreased sharply in postmenopausal stage because of the poor ability of osteogenesis. These results indicate that bone loss was an enduring phenomenon, with osteogenic capability critically weakened.

The effects of MT on bone and bone metabolism were then explored. MT was administered orally at a dose of 100 mg/kg, as in previous studies [20, 28, 32]. It was shown by ELISA that MT reached relatively high concentrations in blood after administration. MT intervention resulted in an increase in bone mass in the sham group, demonstrating that MT can be advantageous in normal rats. It is significant that bone loss was substantially inhibited, while the deterioration of bone microarchitecture was apparently ameliorated with endochondral ossification and primary trabeculae increased in the OVX groups. It was apparent that MT was more effective in the osteoporotic phase compared with the perimenopausal phase according to the observations of micro-CT and HE staining. Serum calcium and phosphorus are generally considered biochemical markers of bone metabolism [33], however, no significant changes were observed in serum calcium and phosphorus compared with each control group. Administration of 100 mg/kg of MT increased levels of serum ALP and OCN in the sham group, demonstrating that MT promoted osteogenesis in normal rats. In the perimenopausal phase, the ability of MT to promote osteogenesis successfully inhibited increased serum ALP caused by bone loss, with a similar trend observed with serum OCN, although not significantly so, as a result of it representing a late marker of osteogenesis. After the perimenopausal period, fluctuations in hormone levels declined and the rats entered a period of continuous bone loss. In the osteoporotic phase, the poor ability to form bone was not sufficient to resist the destruction of the bone by bone resorption, thus, the promotion of osteogenesis by MT increased the levels of serum OCN. Nevertheless, administration of 100 mg/kg of MT enhanced bone mass and regulated bone metabolism in each of the normal, perimenopausal, and clearly osteoporotic phases. However, the concrete mechanisms for this phenomenon is unclear and then, we examined the effects of MT on different time point of osteogenic differentiation.

As described above, OVX weakens osteogenesis in rats, leading to a loss of bone mass. Specifically, OVX has been shown to lead to impaired osteogenic potential of BMSCs [34]. Intervention with MT reversed bone loss in OVX rats and significantly enhanced their osteogenic capability, but a specific explanation or mechanism has so far remained unclear. We hypothesize that MT improves osteogenesis by promoting the osteogenic differentiation of BMSCs in the whole process. Mouse BMSCs were cultured with 100 μmol/L MT as the intervention concentration, supported by the results of a CCK-8 assay, consistent with previous research [35]. We found that 100 μmol/L MT increased the mRNA expression of *Col1a1* and *Runx2* while the mRNA expression of *Alpl*, ALP activity and the extent of ALP staining exhibited no statistical difference three days after intervention compared with a single intervention of osteogenic induction. Seven days after intervention, all indicators were promoted by MT intervention except for ALP activity. In the late stage of osteogenesis, all indicators were promoted by MT intervention except for the mRNA of *Runx2* 14 d after intervention and the extent of ARS staining at the end of osteogenic differentiation was also promoted. Moreover, ARS staining indicated that osteogenesis and matrix mineralization were promoted. The results indicate that MT plays a limited role in the early stage of osteogenesis and plays a more obvious role in the middle and late stages of osteogenesis. Considering the process of osteogenic differentiation and it takes a certain amount of time to accumulate for intervention, we think that MT can promote osteogenesis in the whole process of osteogenic differentiation.

The imbalance between bone formation and resorption might be caused by a number of factors, such as oxidative stress and inflammation [33], considered to be the principal pathogenesis of OP. It has been demonstrated that MT can inhibit osteoclastogenesis and bone resorption through multiple channels [36, 37]. We found that MT not only promoted osteogenesis in normal rats but also reversed bone loss in the perimenopausal and osteoporotic phases, possibly caused by promoting the osteogenic differentiation of BMSCs. Moreover, it has been found that MT can save bone mass through antioxidant and anti-inflammatory effects [19, 38]. These provide evidence that MT can be utilized to treat postmenopausal OP.

There exist a number of limitations to the study. Firstly, although OVX is a classic method to establish a suitable model, replicating the principal characteristics of hypoestrogenism with menopause and postmenopausal OP, the rapid decline in hormones observed in the menopausal transition period was nevertheless not contemplated, and it is not the same as the menopause in its natural state. Secondly, age is inevitably considered in OP studies, while the design of the present study inevitably utilized an 8-week induction period. If surgery had been performed at the same time, rats in late OVX groups would have been 8-week older than the others. If all rats had been anesthetized at the same time, the rats in late OVX groups would have had surgery 8 weeks earlier than the others. Ultimately, the second design was selected, because the OVX rats were not considered to be in a different state as the non OVX rats prior to surgery, although, in reality, there will have been some differences. Finally, although we established that MT could enhance bone mass in normal rats, reverse OP, and promote osteogenesis by enhancing the osteogenic differentiation of BMSCs, the specific mechanisms for the effects of MT remain unknown. Therefore, more investigations are required to elucidate the mechanisms of action of MT.

## Conclusion

Taken together, the study demonstrated that MT enhances bone mass in rats in the natural, menopausal, and postmenopausal phases, possibly due to the promotion of the whole osteogenic differentiation of BMSCs. The results provide evidence that MT would be suitable for use as an anti-osteoporosis drug in the whole menopausal process and might have more effects in postmenopausal phase. Postmenopausal OP is exacerbated over time and, although MT can play a role during the postmenopausal period, it is inevitable that bone loss will continue. The earlier women take MT at the time of menopause, the more beneficial it is for bone mass. Therefore, we suggest that elderly women are prescribed dietary supplements of MT prior to the menopause.

## Data Availability

The datasets used and analyzed during the current study are available from the corresponding author on reasonable request.

## Abbreviations

OP: Osteoporosis
BMD: Bone mineral density
MT: Melatonin
ALP: Alkaline phosphatase
PRMT1: Protein arginine methyltransferase-1
RANKL: Receptor activator of nuclear factor-κB ligand
SIRT1: Silent information regulator type 1
SIRT3: Silent information regulator type 3
OVX: Ovariectomy
BMSCs: Bone marrow mesenchymal stem cells
PFA: Paraformaldehyde
ELISA: Enzyme-linked immunosorbent assay
OCN: Osteocalcin
BV/TV: Bone volume ratio
BS/BV: Bone surface/volume ratio
Tb.Sp: Trabecular separation/spacing
Tb.N: Trabecular number
Tb.Th: Trabecular thickness
Ct.Th: Cortical thickness
CCK-8: Cell counting kit-8
OD: Optical density
ARS: Alizarin Red Staining
